# In-person and virtual adaptation of an interprofessional palliative care communications skills training course for pediatric oncology clinicians

**DOI:** 10.21203/rs.3.rs-3228580/v1

**Published:** 2023-08-11

**Authors:** Karen M. Moody, Clark Andersen, Julie Bradley, Lauren Draper, Timothy Garrington, Jonathan Gill, Douglas Harrison, Masanori Hayashi, Amy Heaton, Cynthia Holladay, Alex Lion, Alakh Rajan, Beatriz Rozo, Daniel Runco, Laura Salvador, Verna Ferguson, Robert Arnold

**Affiliations:** MD Anderson Cancer Center; MD Anderson Cancer Center; University of Colorado Hospital; Cardinal Glennon Children’s Medical Center; University of Colorado Hospital; MD Anderson Cancer Center; MD Anderson Cancer Center; University of Colorado Hospital; MD Anderson Cancer Center; Indiana University; Indiana University; MD Anderson Cancer Center; MD Anderson Cancer Center; Indiana University; MD Anderson Cancer Center; Saint Louis University; University of Pittsburgh

**Keywords:** child, cancer, communication, palliative care, training, clinicians

## Abstract

**Purpose:**

Effective, empathic communication is crucial for pediatric oncology clinicians when discussing palliative and end-of-life (PC/EOL) care with parents of children with cancer. Unfortunately, many parents report inadequate communication at these distressing times. This study evaluates the communication skills training (CST) clinicians received to deliver a PC/EOL communication intervention as part of a multi-site randomized-controlled trial (RCT).

**Methods:**

Clinicians from eight sites formed dyads (one physician and one nurse [RN] or advanced practice provider [APP]) and were trained over 3 days (in-person or virtually). Training was adapted from VitalTalk^™^ and included didactic instruction, videos, visual aids, and dedicated time to practice with simulated patients. Study participants completed a confidential, post-training online evaluation survey. A self-reported quality assurance checklist was used to measure fidelity to the communication protocol when delivered to parents during the RCT.

**Results:**

Thirty clinicians completed training; 26 completed post-training surveys including twelve (46.1%) physicians, 8 (30.8%) RNs and 6 (23.1%) APPs. Most were female (65.4%); white (80.8%), not Latinx (88.5%); 40-50 years old (53.9%); and in practice over 10 years (65.4%). Nine (34.6%) trained in-person; the rest trained virtually. Ninety-two percent reported the course was valuable or very valuable for developing their PC/EOL communication skills and 96% reported learning something new. Dyads trained virtually had similar fidelity to those trained in-person (95% and 90% respectively) when delivering the PC/EOL communication intervention to parents.

**Conclusion:**

This PC/EOL CST was valuable for improving pediatric oncology clinicians’ communication skills, successfully implemented in-person and virtually, and translated effectively into practice.

## Introduction

When a child with cancer cannot be cured, parents are faced with difficult decisions surrounding cancer treatments, and end-of life (EOL) care. Research has shown that parents desire timely, clear and honest prognostic information delivered empathically by their medical team to facilitate EOL decision-making for their child [1]. Parents are significantly less upset by prognostic discussions when physicians convey information in a sensitive manner [2]. Prognostic information should be communicated early and in a stepwise progression. Parents need clinicians to be honest about prognosis and to persevere through the discussion when parents express strong emotions. They want clinicians to leave room for hope, even when prognosis is poor [3]. Parents’ perspectives need to be heard and valued by the medical team and clinicians need to know that their words will have a lasting impact on bereaved families [4]. Unfortunately, many parents report inadequate communication at these most distressing times, reporting specifically having received incomplete medical information and lack of emotional support or ample time to make decisions [5]. In a study of prognostic information provided by pediatric oncologists to parents of children with advanced cancer, three distinct patterns emerged: (a) absent (no information shared); (b) deferred (information shared only at the very end-of life); and (c) seed planting (hinting at incurability over time) [6]. These findings suggest a significant need for improvement and standardization in communicating prognosis, goals of care (GOC) and benefits of PC/EOL support with affected parents.

Previous research has pointed to several reasons behind delayed or inadequate communication delivered by clinicians [7, 8]. The lack of training is found in most studies to be a problem. In one survey, 110 pediatric oncology fellows reported: 32% received formal poor-prognosis and PC/EOL CST during their fellowship; 27% received no PC/EOL CST during medical school, residency or fellowship; and 21% did not receive any feedback on their delivery of PC/EOL communication skills [9]. Most pediatric oncologists’ CST includes only informal apprenticeships (observations, role modeling) often without feedback, as opposed to recommended communication-focused, experiential-learning activities (role-playing with standardized patient actors; constructive debriefing feedback) [8].

Pediatric-oncology nurses (RNs) and advanced practice clinicians (APPs) also report PC/EOL communication-related challenges. They struggle with determining their role in participating in prognostic-related communication with families and often have received no or limited PC/EOL CST [10]. Pediatric oncology RNs and APPs report not being included and/or being informed when physicians have met with parents to discuss prognosis-related information. Fortunately, data support that prognostic-related CST enhances their confidence and skill as well [11].

The Institute of Medicine Report on Delivering High Quality Cancer Care recommended comprehensive and formal CST for cancer care teams about prognosis and palliative care, and to integrate visual decision aids to personalize information at key decision points along the continuum of the patient’s cancer trajectory [12].

In light of national recommendations to address recognized gaps in physician and RN/APP training in prognostic-related communication and parent-identified communication needs, our study team developed a palliative care (PC)/EOL communication intervention and training course to assist pediatric oncologists, RNs and APPs to discuss prognosis and GOC as an interprofessional team with parents of children with cancer who have less than 50% chance of survival. We developed a prospective-cluster-randomized-trial to test our PC/EOL communication intervention on children’s EOL outcomes and patient-reported quality of life (QOL) (parent and child) “The Informational Meetings for Planning and Coordinating Treatment” (IMPACT) study. This manuscript provides a description of our PC/EOL communication-intervention training courses (in-person and virtual) and the post-training evaluation results completed by our clinicians.

## Methods

We recruited pediatric oncologists RNs and APPs to form a dyad of one physician and one RN or APP from 8 institutions: MD Anderson Cancer Center (TX), Children’s Hospital Colorado (CO), Children’s Hospital of Atlanta (GA), SSM Health Cardinal Glennon Children’s Hospital (MO), Riley Hospital for Children (IN), Seattle Children’s Hospital (WA), Children’s Wisconsin (WI), and Nemours Children’s Hospital (DE). The dyads were randomized to the intervention or an attention-control group by clinician practice (non-central nervous system solid tumor or neuro-oncology); size of practice (large or medium); and whether the clinicians had previous formal PC/EOL CST. Dyads received 3days of training (in-person or virtually). In-person training occurred at Bradford Woods, a 2500-acre retreat center of Indiana University in November 2019. Dyads recruited subsequently and unable to participate in-person due to the COVID19 pandemic were trained virtually. There were 5 virtual trainings between July 2020 and January 2023. Virtual training was adapted from in-person training and used the same agenda, didactic presentations, videos and case-based role plays with simulated actors.

**Figure 1:**
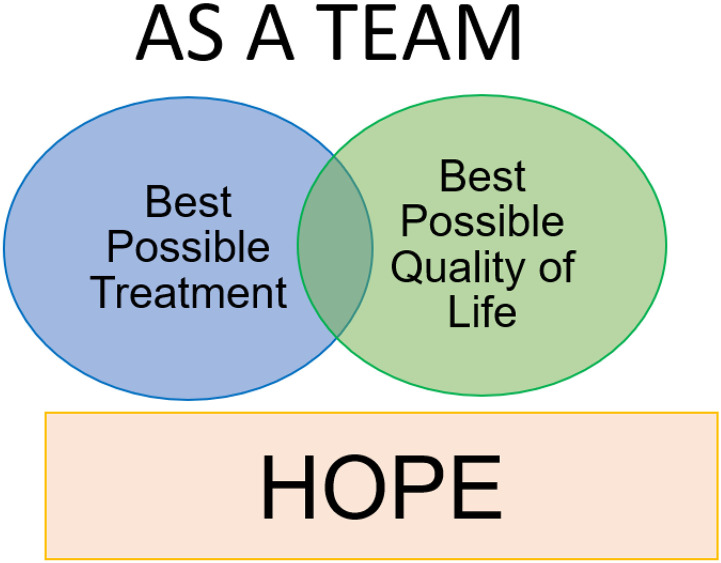
Diagram I

**Figure 2:**
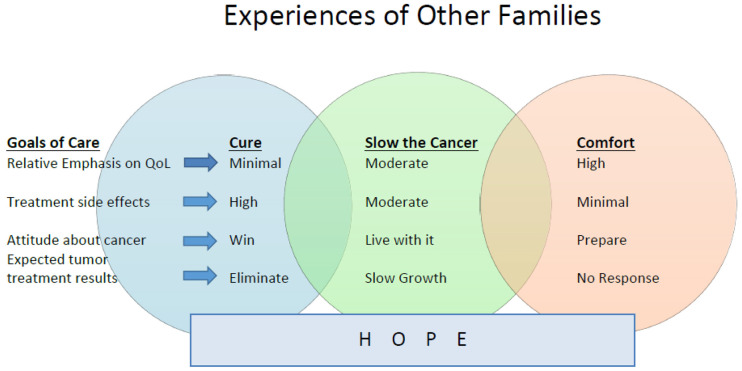
Diagram II

Our PC/EOL communication intervention consisted of a conversation guide based on VitalTalk^™^ REMAP [10] modified for use at different timepoints along the continuum of care with parents of children with cancer. Table 1. In addition, two novel features incorporated into the intervention were the use of dyads to co-deliver the intervention instead of single clinicians, and the development of two visual aids (Diagrams I and II) to use alongside the guide. Diagram 1 depicted hope, dual GOC (QOL and best-possible treatment) and team support with two overlapping circles. See Fig. 1. Diagram II illustrated 3 GOC: “cure the cancer”, “slow the cancer”, and “comfort” with 3 overlapping circles. See Fig. 2.

Our training method was based on the VitalTalk^™^ course with dyads trained as a team and interprofessional team principles added to the curriculum. Evidence-based VitalTalk^™^ skills such as “Ask-Tell-Ask”, silence after giving bad news, “I wish/I worry” statements, and expressive use of empathy described by the acronym NURSE (naming, understanding, respecting, supporting, exploring) were taught via didactic PowerPoint presentations to learners [13]. The curriculum included dedicated time to practice using diagrams I and II with simulated patients. VitalTalk^™^ videos depicting difficult conversations between patients, family members, RNs, APPs, and doctors were shown and the unique roles of interprofessional team members were emphasized in the training. Sample videos were created with the VitalTalk^™^ facilitators (LV, JH, KM) portraying the dyad with a simulated patient/parent/family member for the specific pediatric oncology scenarios of breaking bad news, discussing GOC at time of disease progression, and EOL planning at the terminal stage of disease. These videos served as resources and “Booster Training” for participants to review as needed throughout the study.

Four pediatric-oncology cases were created from the experience of one of the MPIs (KM) and literature review. Each case depicted a child with a poor cancer prognosis and included three different time points: new diagnosis, progression of disease, and approaching end of life. Actors were hired to portray an adolescent patient (in one case), parent(s), and family members. The actors were given extensive backgrounds about their illness, their mindset, family dynamics and their cultural context. During actor-training, actors were given time to practice their roles with the facilitators (KM, LV, EN). A key component of the actors’ job was to respond realistically when the clinicians presented the PC/EOL intervention. For example, if the dyad partners did not exhibit empathy when giving poo prognostic information the actors might respond by disengaging from the conversation or becoming frustrated [13].

Each dyad was assigned to a simulated patient and family member(s) to practice the communication intervention in the context of the 3 clinical time points. Dyads role-played with the same family over the 3-day course to simulate a longitudinal relationship. A brief synopsis of the case (including key medical and social details of the patient and their parent[s] and family member[s]) was given to the dyad in temporal order, immediately prior to each role play. During the role plays, the other dyads watched as one dyad performed the communication intervention and then provided feedback about what the dyad did well. This process enabled participants to learn from each other and observe multiple examples of the intervention delivery.

If learners felt stuck in their interactions or the facilitators sensed the learners needed assistance, a “Time out” was called by the learner or facilitator, respectively. During the “Time out” the learning dyad could “phone a friend” or ask observers for suggestions for what to say or do to move the conversation forward. To ensure standardized and consistent delivery of our PC/EOL communication intervention during the study period, dyads were trained to perform quality assurance (QA) on themselves. This QA process required the dyads to fill out a check-list documenting which tasks they did and did not complete during the intervention and included items such as “used NURSE statements” and “reviewed Diagram I with family”. If the dyad did not complete the checklist within 48 hours of the intervention, they would have to complete the check list after listening to the intervention’s audio recording (all intervention sessions were audio recorded). Study intervention fidelity was operationalized as the percentage of tasks completed during the intervention session, with 0 percent = none and 100 percent = all.

A post-training survey included closed-ended demographic questions and questions regarding the value of several aspects of the course using 5-point Likert-like scale, with 5 representing the most favorable response. Four open-ended questions queried opinions on the learning environment (virtual and in-person); novel concepts learned; general course feedback; and how participants wanted to improve their skills when they returned to work. Participants were asked how committed they were to make this change. The survey was sent electronically and anonymously to the participants. An informed consent statement was included in the invitation email. Participants were invited to read the informed consent statements and proceed to the survey study if they agreed with them. The MD Anderson Cancer Center IRB approved the post-training survey study in accordance to the U.S. Department of Health and Human Services regulations for the protection of human subjects in research. The Indiana University single IRB approved the IMPACT study in accordance to the U.S. Department of Health and Human Services regulations for the protection of human subjects in research. QA data is presented to illustrate intervention fidelity during the study to date.

## Data Analysis

Descriptive statistics were used to describe the frequencies, median (M), and interquartile range (IQR), of the learner’s responses to the closed-ended survey questions. Manual qualitative analysis on the open-ended text responses was performed by K.M. Text responses were de-identified and reviewed for common themes and patterns. Responses were classified into categories based on similarities and differences within and across questions. Frequencies of categories were calculated. Notable quotes for categories were added for context. Categories were incorporated into themes, where appropriate [14]. A second author (AH) manually reviewed the qualitative data and resulting categories and themes to reduce bias. Differences in qualitative data analysis results were discussed among the two reviewers until reaching consensus.

## Results

Thirty clinicians completed training and 26 completed post-training surveys with representation from 6 of the 8 sites. Twelve (46.1%) of the sample with completed surveys were physicians, 8 (30.8%) were RNs and 6 (23.1%) were APPs. Most of the sample identified as female (65.4%); white (80.8%) not Hispanic or Latinx (88.5%); 40–50 years old (53.9%); and in practice over 10 years (65.4%). Nine participants (34.6%) trained in-person; the rest trained using the virtual platform. Table 2.

Survey responses were favorable with 100% of participants rating the overall value and quality of the training education content as 4 or 5, with median (M) and interquartile range (IQR) of (M = 5; IQR 4,5). The facilitator quality was rated 4 or 5 by 96% of participants (M = 5; IQR 4,5). Nighty-six percent rated 4 or 5 for the value of the session on adapting the intervention to the clinical environment (M = 5; IQR 4.5) as well as interprofessional education components (M = 4.5; IQR 4,5). The value of VitalTalk^™^ – skills (e.g. Ask-Tell-Ask, I wish/I worry, NURSE, etc.) were rated 4 or 5 by 93% of participants, (M = 5; IQR 4,5). Ninety-two% of participants rated 4 or 5 for value of: a) the training program to the development of PC/EOL communication skills, (M = 4; IQR 4,5); b) the adapted REMAP guide (M = 4.5; IQR 4,5); c) training as a dyad, (M = 5; IQR 4.25,5); and d) Diagram II: “Experience of other families” (M = 5; IQR 4.25,5). Eighty-eight% of participants rated 4 or 5 for the value of Diagram I: “As a Team” (M = 4.5; IQR4,5) and the value and quality of role play (M = 5; IQR4,5). The quality of power point presentations, written materials, and videos were rated 4 or 5 by 77%, 81% and 89% of participants, respectively with (M = 4; IQR4,5) for each. Booster training was rated by 71% as 4 or 5 (M = 4; IQR3,5.) Eight of 9 survey participants thought going to Bradford Woods for in-person training was worth the time and effort since it allowed for dedicated time and space, bonding of the participants, and facilitated practicing communication skills and intervention with simulated-actor patients.

**Figure 3:**
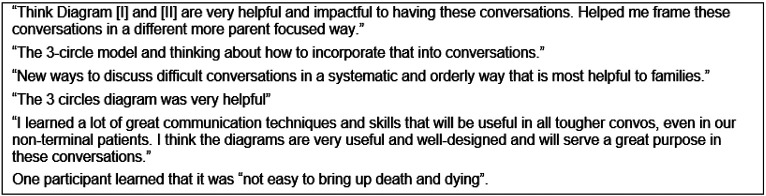
Quotes regarding newly learned information

Ninety-six percent of learners reported learning something new during training. Open-ended comments revealed 6 specific categories of new information learned, including: how to have difficult conversations; VitalTalk^™^ phrases for during difficult conversation; the helpfulness of both observing and participating in role-play to improve communication skills; importance of interprofessional care and how to work effectively in an interprofessional dyad; the utility of using Diagrams 1 and 2 during difficult conversations, especially Diagram II; and how research and data collection works. The visual aids were the most cited category of new learning, noted by 7 (27%) participants. Comments regarding new information learned are shown in Fig. 3.

Participants who trained online were asked to comment on their experience. The overall theme that emerged from the participants’ comments of participants who took part in virtual training was that virtual training was adequate towards meeting the goal of improving palliative care communication skills. This was particularly true for clinicians that were conducting virtual visits at their sites. For example, one participant stated, “As we have implemented video visits in true clinical practice, I do not feel the training with mock patients in this venue was hindered.”

Six of 17 virtual participants (35.3%) thought that in-person training would have been better. Reasons cited for this included: greater ability to engage in nonverbal communication, in-person training would have felt more realistic, and in-person training provides increased interaction among participants. Participants reported on the benefits of avoiding travel (i.e. saving time and money). A suggestion for improving virtual training was to have all of the actors in one [Zoom] room (window).

**Figure 4:**
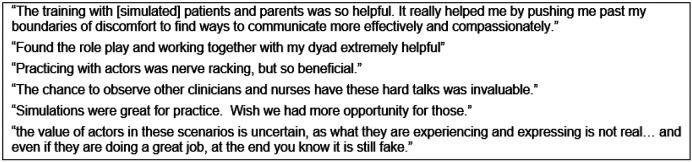
Quotes regarding role-play

Participants were given free-text space to provide general course feedback. The theme of these responses was the overall format and tools were highly valuable and the 3-day time frame was appropriate. Mirroring the quantitative data, participants mostly found role-play with actors to be beneficial for honing communication skills and allowing for a safe space to practice CST. Some clinicians asked for more role-play; however, 2 clinicians expressed a general dislike for role-play with simulated patients. Quotes regarding role-play are shown in Fig. 4.

**Figure 5:**
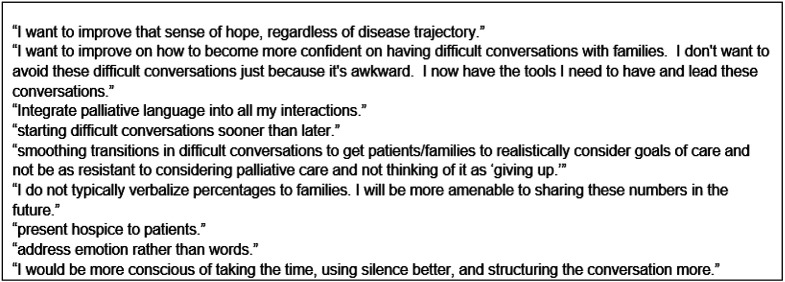
Quotes regarding practice changes

The majority (95.6%) of clinicians were committed to making changes in their practice as a result of training. Specifically, the learners identified active listening, expressing empathy, being curious, identifying patients’ broad goals and using silence as the specific skills they could incorporate into their practice. Sample quotes regarding practice changes are shown in Fig. 5.

As of May 2023, 22 (85%) trained clinicians have delivered 62 audio-recorded PC/EOL communication interventions to the parent(s) of 29 children with a poor cancer prognosis on our IMPACT study. The median self-assessed fidelity of completed protocol-communication tasks is 92.5% with a range from 65 to 100%. Dyads trained virtually had similar fidelity to those trained in-person (95% and 90% respectively).

## Discussion

A new openness to using percentages in prognostic discussions

In this report, we describe the post-training evaluation results of pediatric oncology clinicians that were trained to deliver a PC/EOL communication intervention to parents of children with a poor cancer prognosis. We incorporated published recommendations for CST for pediatric oncologists and RNs/APPs for having PC/EOL discussions with parents of children with a poor-prognosis brain tumor (i.e., use of trained actors, interprofessional education, 3-day training) [15], VitalTalk^™^ teaching methods [16] and visual aids that we created to facilitate shared-decision making [1, 12]. The resulting interprofessional PC/EOL course was well received, highly rated for both quality and value, and included all key recommendations from the 2018 American Society for Clinical Oncology consensus guideline on patient-clinician communication (i.e. incorporation of validated techniques, supervised skills practice, real time feedback, exercises that enhance empathy; increase self-awareness and situational awareness related to emotions, attitudes and beliefs; and use of trained facilitators) [17]. A notable finding was that even learners with significant years of pediatric oncology experience expressed gaining new knowledge and acquiring new skills (utility of visual aids; how to hold difficult PC/EOL conversations, and interprofessional team delivery). Our learners were highly committed to incorporate new skills and knowledge into their practice. These findings (high value ratings, improved learner confidence, skills, and motivation for change) are consistent with other PC/EOL CST courses for adult primary care and subspecialty physicians and nurses [16].

After our training course, nearly all participants had an opportunity to apply their new skills with parents and achieve acceptable intervention-fidelity ratings. The visual aids were reported as particularly valuable features of the intervention as they helped to frame and structure the conversation. Another unique aspect of our approach was training our clinicians in interprofessional dyads. It is possible that by training dyads to use the communication plan, clinicians felt more secure in these conversations as their partner was there to assist if one of them had a loss for words or needed help supporting the parents emotionally.

While we intended for this training to occur in person, the COVID19 pandemic quarantines required us to convert our training program to an alternative virtual format for 65% of participants. Although the virtual format did not allow for participants to interact to the same degree as the in-person format, it was effective in its aim to train the learners in using the communication plan successfully. The virtual format further allows for a potential large-scale implementation going forward. Despite the need for a considerable investment of time, the participants thought the course was worth the time and effort and would recommend it to their colleagues. These findings are consistent with the results from a systematic review of CST courses in pediatric oncology which reported uniformly positive endorsements by the participants [18].

## Conclusion

This report presents a detailed description of a highly rated pediatric oncology interprofessional PC/EOL CST that was also successfully adapted to a virtual environment. Currently, we are collecting outcomes on children and parents receiving the communication intervention compared to an attention-control group on the IMPACT trial. If the children show improved quality of life at end of life and parents have more hope and less distress through their receipt of the intervention, we will develop an implementation grant to broadly disseminate this training to pediatric oncology clinicians nationwide.

## Figures and Tables

**Table 1.** REMAPS / COMPLETE Intervention

	Strategy	Key Elements
**R**	RAPPORT / REVIEW / REFRAME	Ask family how they are doing currently Ask-Tell-Ask: Diagnosis Ask-Tell-Ask: Treatment Ask-Tell-Ask: Prognosis (using percentages) Review Diagram I and II with family
**E**	EXPECT EMOTION	Provide realistic hope messages Provide non-abandonment, “we” messages Use VitalTalk NURSE Statements (Naming, Understanding, Respecting, Supporting, Exploring)
**M**	MAPPING / MEANING	Ask what matters most to the child/family (values) Ask about the child/family’s hopes, worries, fears, concerns Reflective Listening
**A**	ALIGN GOALS	Review Diagram I: Experiences of Other Families Introduce concept of goals of care Establish specific goals of care for child and family Demonstrate sensitivity to the fact that families have dual goals of care (i.e. cure & comfort)
**P**	PLAN	Discuss treatment options consistent with child’s prognosis and family’s goals of care Determine plan for moving forward based on goals of care, values & hopes Discuss recommendations for treatment (i.e. cancer directed therapy, antibiotics, transfusions, etc) Introduce, explain, and discuss parental preferences for breathing machines and CPR/DNR
**S**	SYNOPSIS / SUMMARY	Ask child/family if they have any questions Express gratitude to child/family Reassure presence and support of clinical team Give slides to family for future reference Plan next visit

**Table 2:** Demographics

Measure	Item	Count	Percentage (%)
	20–30 years	1	3.8
**Age**	30–40 years	9	34.6
	40–50 years	14	53.9
	> 50 years	2	7.7
**Gender (selected choice)**	Female	17	65.4
	Male	9	34.6
	White	21	80.8
**Race**	Asian	3	11.5
	Other	2	7.7
**Ethnicity**	Latino or Hispanic	3	11.5
	Not Latino or Hispanic	23	88.5
	Advanced Practice Nurse (APN)	6	23.1
**Current Position**	Clinic Registered Nurse	8	30.8
	Physician (MD or DO)	12	46.1
**Specialty within**	Neuro oncology	7	26.9
**Pediatric Oncology**	Non-Neuro Solid Tumor	14	53.9
	Other	5	46.2
**Years of Hospital**	Less than 1 year	1	3.8
**Experience in Pediatric**	1–5 years	6	23.1
**Oncology Specialty**	5–10 years	2	7.7
	Over 10 years	17	65.4
**Previous Palliative Care**	Yes	12	46.1
**Communication Training?**	No	14	53.8

## References

[1] Xafis V., Wilkinson D., & Sullivan J. (2015). What information do parents need when facing end-of-life decisions for their child? A meta-synthesis of parental feedback. BMC Palliative Care, 14(1), 1–11. 10.1186/s12904-015-0024-0 25924893 PMC4424961

[2] Mack J. W., Wolfe J., Grier H. E., Cleary P. D., & Weeks J. C. (2006). Communication about prognosis between parents and physicians of children with cancer: parent preferences and the impact of prognostic information. Journal of Clinical Oncology, 24(33), 5265–5270. 10.1200/jco.2006.06.5326 17114660

[3] Robert R., Razvi S., Triche L. L., Bruera E., & Moody K. M. (2022). Bereaved Parent Perspectives on End-of-Life Conversations in Pediatric Oncology. Children, 9(2), 274.4. 10.3390/children9020274 35204993 PMC8870516

[4] Contro N., Larson J., Scofield S., Sourkes B., & Cohen H. (2002). Family perspectives on the quality of pediatric palliative care. Archives of Pediatrics & Adolescent Medicine, 156(1), 14–19. 10.1001/archpedi.156.1.14 11772185

[5] Aschenbrenner A. P., Winters J. M., & Belknap R. A. (2012). Integrative review: parent perspectives on care of their child at the end of life. Journal of pediatric nursing, 27(5), 514–522. 10.1016/j.pedn.2011.07.008 22920662

[6] Kaye EC, Stall M, Woods C, Velrajan S, Gattas M, Lemmon M, Baker JN, Mack JW. (2021). Prognostic communication between oncologists and parents of children with advanced cancer. Pediatrics, 147(6). Advance online publication. 10.1542/peds.2020-044503 PMC8503785 33952691

[7] Mack J. W., & Joffe S. (2014). Communicating about prognosis: ethical responsibilities of pediatricians and parents. Pediatrics, 133(Supplement_1), S24–S30. 10.1542/peds.2013-3608e 24488537

[8] Feraco A. M., Brand S. R., Mack J. W., Kesselheim J. C., Block S. D., & Wolfe J. (2016). Communication skills training in pediatric oncology: moving beyond role modeling. Pediatric Blood & Cancer, 63(6), 966–972. 10.1002/pbc.25918 26822066 PMC5861499

[9] File W., Bylund C. L., Kesselheim J., Leonard D., & Leavey P. (2014). Do pediatric hematology/oncology (PHO) fellows receive communication training? Pediatric Blood & Cancer, 61(3), 502–506. 10.1002/pbc.24742 24039096 PMC5561546

[10] Childers J. W., Back A. L., Tulsky J. A., & Arnold R. M. (2017). REMAP: a framework for goals of care conversations. Journal of Oncology Practice, 13(10), e844–e850. 10.1200/jop.2016.018796 28445100

[11] Newman A. R., Callahan M. F., Lerret S. M., Oswald D. L., & Weiss M. E. (2018). Pediatric oncology nurses’ experiences with prognosis-related communication. Oncology Nursing Forum, 45(3), 327–337. 10.1188/18.onf.327-337 29683123

[12] Institute of Medicine. (IOM) 2013. Delivering High-Quality Cancer Care: Charting a New Course for a System in Crisis. Washington, DC: The National Academies Press. 10.17226/18359 24872984

[13] Back AL, Arnold RM, Tulsky JA, Baile WF, Fryer-Edwards KA. Teaching communication skills to medical oncology fellows. J Clin Oncol. 2003 Jun 15;21(12):2433–6. 10.1200/jco.2003.09.073 12805343

[14] Applied Qualitative Research Design: A Total Quality Framework Approach (Roller & Lavrakas, 2015, pp. 262–263).

[15] Hendricks-Ferguson V.L., Kane J.R., Pradhan K., Chie-Schin S., Gauvain K., & Haase J. (2015). Evaluation of a physician and nurse dyad training procedures to deliver a palliative and end-of-life communication intervention to parents of children with a brain tumor. Journal of Pediatric Oncology Nursing, 32(5), 337–347. 10.1177/1043454214563410 25623029 PMC5918283

[16] Back AL, Fromme EK, Meier DE. Training Clinicians with Communication Skills Needed to Match Medical Treatments to Patient Values. J Am Geriatr Soc. 2019 May;67(S2):S435–S441. doi: 10.1111/jgs.15709. 10.1111/jgs.15709 31074864

[17] Gilligan T., Bohlke K., & Baile W. F. (2018). Patient-clinician communication: American Society of Clinical Oncology consensus guideline summary. Journal of Oncology Practice, 14(1), 42–46. 10.1200/jop.2017.027144 28915077

[18] Kaye EC, Cannone D, Snaman JM, Baker JN, Spraker-Perlman H. The state of the science for communication training in pediatric oncology: A systematic review. Pediatr Blood Cancer. 2020;67:e28607. 10.1002/pbc.28607 32706453

